# The relationship between physical fitness and drop vertical jump biomechanics in male college basketball players

**DOI:** 10.7717/peerj.20613

**Published:** 2026-02-02

**Authors:** Liang Guo, Kaiyuan Qu, Jing Zhang, Yufeng Zhang, Zhiye Zhang, Ying Wu, Dan Wang

**Affiliations:** 1 School of Physical Education & Sports Science, South China Normal University, Guangzhou, China; 2 School of Athletic Performance, Shanghai University of Sport, Shanghai, China; 3 Department of Obstetrics, Guangdong Maternal and Child Health Care Hospital, Guangzhou, China; 4 Shanghai Research Institute of Sports Science, Shanghai, China

**Keywords:** Vertical jump ability, Sports biomechanics, Lower limbs, Joint

## Abstract

**Background:**

Studies have shown that vertical jump biomechanics or patterns may be related to physical fitness. This study investigated the relationship between physical fitness and drop vertical jump (DVJ) biomechanics in male college basketball players.

**Methods:**

Health-related physical fitness was measured by 20s sit-up, core endurance and flexibility test; whilst athletic-related physical fitness by Y-balance test and dominant extremity single-leg hop distance test. Kinetics and kinematics during DVJ were evaluated by VICON.

**Results:**

Five-level side bridge correlated negatively with angle displacement of hip adduction (*p* = 0.014) and positively with moment of knee flexion (*p* = 0.033); 8-level abdominal bridge correlated positively with moment of knee flexion (*p* = 0.01); ankle dorsiflexion range of motion correlated negatively with mediolateral ground reaction force (*p* = 0.025), angle displacement of knee flexion (*p* = 0.004), moment of ankle plantarflexion (*p* = 0.009), and positively with angle displacement of ankle plantarflexion (*p* = 0.015); ankle plantarflexion ROM correlated negatively with angle of knee flexion (*p* = 0.012); trunk flexion ROM correlated negatively with moment of ankle plantarflexion (*p* = 0.014).

**Conclusion:**

Health-related physical fitness could be the alternatives for DVJ biomechanics assessment.

## Introduction

Vertical jump, as a vital skill, is routinely performed in various sports (Ford et al., 2017; Feltner, Fraschetti & Crisp, 1999), like volleyball (Xu et al., 2022), basketball, and soccer (Karatrantou et al., 2019). The blocking in volleyball, rebounding in basketball, and the header in soccer require high levels of vertical jump ability (Karatrantou et al., 2019). In men’s basketball games, the athletes perform approximately 40 to 50 vertical jumps, encompassing movements such as rebounding, blocking, and shooting (Cabarkapa et al., 2024). A decline in physical fitness, whether from inadequate training or fatigue, can impair vertical jump biomechanics (Gathercole, Sporer & Stellingwerff, 2015; McLellan, Lovell & Gass, 2011b). Compromised landing mechanics, in turn, increase the risk of lower limb injuries by altering joint kinematics and kinetics (Jie et al., 2024; Xu et al., 2024; Xu et al., 2023). While three-dimensional (3D) motion analysis system and force platforms are used for biomechanical analysis of vertical jump movements (Ford et al., 2017; Sasaki et al., 2019; Moran & Wallace, 2007), its requirement for expensive equipment, specific laboratory space, and time-consuming setup limits its practical application for coaches in the field (Mobini, Behzadipour & Saadat, 2015). Thus, it is imperative to find a valid alternative assessment for convenient implementation in practice.

Sports performance is influenced not only by technical skill but also by physical fitness, encompassing both health-related and skill-related components (Puszczałowska-Lizis et al., 2025). As a kind of sports performance, the vertical jump can also be affected by physical fitness. Studies have shown that vertical jump biomechanics or patterns may be related to core strength (Guo, Wu & Li, 2020), core endurance (Sasaki et al., 2019; Santos et al., 2019), flexibility (Opplert & Babault, 2018; Howe et al., 2019), balance (Howe et al., 2019), and lower extremity power (Wilczynski et al., 2021). Core strength and endurance are directly related to an athlete’s ability to stabilize their trunk during dynamic movements like jumping (Cao et al., 2024). Weak or fatigued core muscles negatively affect an athlete’s jumping mechanics, leading to suboptimal force transfer and reduced jump height. Endurance in the core muscles allows for better posture control during the countermovement and the explosive push-off phase of a jump (Guo et al., 2021). Range of motion (ROM), particularly in the hips, knees, and ankles, allows athletes to optimize their jump mechanics (Katircioglu et al., 2025). Greater flexibility allows for a deeper countermovement (or squat) during the preparatory phase of the jump, which in turn helps to generate more force during the explosive phase (Macrum et al., 2012). Balance is crucial for vertical jump biomechanics because it ensures proper body alignment during both take-off and landing (Ibrahim et al., 2025). The landing phase of a vertical jump requires strong dynamic balance to maintain postural control and prevent collapse of the lower limb joints (ankles, knees, hips) (Ibrahim et al., 2025). Single leg hop test measures explosive power, particularly in the dominant leg (Maulder & Cronin, 2005). This is crucial because vertical jump biomechanics heavily relies on the athlete’s ability to generate force quickly and efficiently through their lower limbs (McLellan, Lovell & Gass, 2011a). Research has shown a strong correlation between hop distance and vertical jump height, as both movements require the rapid production of power from the quadriceps, hamstrings, and calf muscles (Maulder & Cronin, 2005). Maulder and Cronin suggested that single leg hop tests were predictive of bilateral vertical jump biomechanics because they assessed unilateral strength, which contributed to the force output during takeoff (Maulder & Cronin, 2005).

Core strength, core endurance, and flexibility correspond to the muscle strength, endurance, and flexibility of health-related physical fitness, respectively (Franklin, Whaley & Howley, 2010). Balance and lower extremity power correspond to the balance and power of skill-related physical fitness, respectively (Franklin, Whaley & Howley, 2010). Guo, Wu & Li (2020) found significant correlations between dynamic core flexion strength and vertical jump height. Sasaki et al. (2019) found that eight weeks of core muscle training (*e.g.*, bench, sideways bench, and Nordic hamstrings) could increase the trunk flexion angle and reduce the angle of knee valgus during a drop jump and single-leg squat. It is speculated that fitness tests might be related to vertical jump biomechanics and could be utilized to assess the landing patterns.

In Chinese sports biomechanics field, 8-level abdominal bridge, 6-level supine bridge and 5-level side bridge are currently employed for core strength and endurance assessment. Differing from conventional core tests like the plank, these protocols quantify strength and endurance while dynamically challenging stability by comprehensively evaluating abdominal, hip, gluteal, back and limb stability across coronal and sagittal planes (Zhao, 2023). They require participants to sustain their joints in a functionally anatomical position in the core region while the supporting base is reduced (Lee, Wang & Zhang, 2024). Twenty seconds sit-up test (20 s-SU) further evaluates trunk flexion strength and endurance. This study incidentally compared these four assessments to determine which demonstrates the greatest association with landing biomechanics during DVJ. The inclusion of hip, knee, ankle, and trunk ROM assessments is due to their critical biomechanical roles in optimizing performance and mitigating injury risk. Adequate ankle dorsiflexion ROM is essential for permitting sufficient anterior translation of the knee during the landing phase, facilitating greater force production while concurrently reducing compensatory movements like knee valgus that increase anterior cruciate ligament (ACL) loading during landing (Bell, Padua & Clark, 2013; Hewett et al., 2005; Fu et al., 2025). Hip mobility, particularly flexion and internal rotation, is fundamental for maintaining pelvic stability and proper trunk positioning; restrictions can lead to aberrant lumbar motion, impair force transfer through the kinetic chain, and contribute to dynamic lower extremity valgus (Bagwell et al., 2016). Finally, adequate trunk ROM and control, particularly in the sagittal plane, are essential both for achieving optimal posture during propulsion and for stabilizing the body during the high-impact landing phase of the DVJ (Kibler, Press & Sciascia, 2006). Collectively, these ROM measures identify potential kinetic chain impairments that can compromise DVJ execution, efficiency, and safety. Furthermore, athletes with higher scores in the Y-balance test (YBT) tend to have better dynamic control, leading to more stable and controlled landings, reducing injury risk (Plisky et al., 2021). Moreover, the YBT is reliable, with a high intraclass correlation coefficient (ICC) ranging from 0.80 to 1.00 (Zheng et al., 2024). The selection of the dominant single-leg hop for distance (D-SLHD) is justified by two key rationales. Firstly, as the primary force-generating limb in the kinetic chain, the dominant leg’s SSC efficiency directly determines landing stability in DVJ (Sammoud et al., 2024). D-SLHD quantifies reactive strength (hop distance) of the dominant limb, predicting DVJ jump height (*r* = 0.91) and ground contact time (Waldhelm & Li, 2012; Reid et al., 2007).

Therefore, this study aimed to investigate the association between physical fitness and vertical jump biomechanics to provide a simpler method for assessing vertical jump ability and landing patterns. We hypothesized that specific components of health-related physical fitness and skill-related physical fitness would correlate with kinetics and kinematics measured during the landing phase of the DVJ. Specifically:

(1) Poor core strength and endurance would be associated with impaired trunk control (*e.g.*, excessive trunk flexion/lateral flexion) and aberrant lower extremity joint kinematics (hip, knee, ankle angles/displacements) during landing.
(2) Restricted ankle dorsiflexion ROM would be associated with reduced ankle dorsiflexion angle and increased compensatory knee movements (*e.g.*, greater knee valgus) during landing.
(3) Restrictions in hip, knee, and/or trunk ROM (*e.g.*, reduced joint flexion angles) would be associated with decreased power capacity and altered kinetics in the lower limbs during landing.
(4) Poor dynamic balance would be associated with reduced postural stability (aberrant joint angle) during the landing phase.
(5) Weak lower extremity power (indicated by D-SLHD distance) would be associated with impaired impact control (*e.g.*, higher peak vertical ground reaction force) during landing.

## Materials & Methods

This was a cross-sectional laboratory-based study designed to investigate the correlations between a battery of field-based physical fitness tests and biomechanical variables collected during the DVJ task. The recruitment of participants for the study commenced on June 9th, 2021, and concluded on September 9th, 2021, spanning a total duration of 3 months. All tests were conducted in our Sports Biomechanics Laboratory over two non-consecutive days by trained members of the research team to ensure consistency. Physical fitness tests were conducted on the first day, followed by the DVJ test on the second day. The experimental temperature was controlled at 18–22 °C. Prior to testing, all participants arrived at the laboratory before 4:00 pm and wore shoes and completed a 10-minute warm-up. The participants wore their daily sports shoes to participate in all the test sessions and did not change their shoes. We required that the shoe models should be closed-type sports shoes. This move aims to simulate the gait performance in real scenarios and improve ecological validity. The warm-up included exercises such as the bench (alternate legs and one leg lift and hold); sideways bench (raise & lower hip, with leg lift); single-leg stance (hold the ball, throw the ball to a partner); squats (with toe raise, walking lunges, one-leg squats); jumping (vertical jumps, lateral jumps, box jumps). The researcher demonstrated each movement, and participants practiced until they were comfortable with them, typically requiring two or three attempts. After the tests, participants followed a cool-down routine to help reduce potential muscle soreness. All tests were conducted in a standardized order to minimize fatigue effects: flexibility tests (trunk, hip, knee, ankle ROM) were performed first, followed by YBT, D-SLHD, and finally core endurance tests (20 s- SU, bridge tests) and DVJ test. The cool-down exercises included quadriceps stretching, hamstring stretching, triceps surae stretching, wall chest stretching. Each testing session lasted approximately 1 h. Participants were permitted to hydrate prior to the tests but rehydration was not allowed during tests to prevent any influence on performance outcomes. The ethics review board of South China Normal University approved the study, which involved human participants (SCNU-SPT-2021-002). Prior to testing, participants would be provided with an informed consent form. All participants would be required to review and sign the consent form before participating in the testing.

### Participants recruitment

*A priori* power analysis was conducted using G*Power 3.1.9.7 with an exact test for bivariate normal correlation (two-tailed) to determine the minimum sample size required. With a significance level (*α*) of 0.05, statistical power of 0.80, and an anticipated population correlation coefficient (*ρ*) of 0.606 (coefficient of determination *ρ*^2^ = 0.367 McCurdy et al., 2014), the analysis indicated a required total sample size of 18 participants. Eighteen amateur male college basketball players (21.9 ± 0.5 yrs, 65.6 ± 1.6 kg, 174.2 ± 2.2 cm, 21.6 ± 0.8 kg/m^2^) were recruited (all left lower extremity dominant) using flyers and in-class announcements from the South China Normal University. Participants were eligible for the study if they met all the following conditions: (1) male collegiate basketball players aged between 18 and 26 years; (2) actively participating in a structured university-level basketball training program for a minimum of 2 consecutive years prior to the study, with a training frequency of no less than 3 sessions per week, each lasting over 1 h. Participants were excluded from the study if they met any of the following conditions: (1) any history of surgery or fracture in the lower back or lower extremities within the previous 12 months; (2) any acute musculoskeletal pain, ligament sprain, or other injuries in the lower back or lower extremities that could affect jumping or landing performance at the time of testing; a diagnosed neurological or vestibular disorder that affects balance; (3) any systemic or chronic disease (*e.g.*, rheumatoid arthritis) known to influence joint function or physical performance; regular use of medication or supplements that could significantly affect neuromuscular performance or pain perception. This study examined the relationships between physical fitness tests (as independent variables) and biomechanical metrics collected during the DVJ task (as dependent variables).

### Method details

#### Health-related physical fitness test

##### Core endurance test. 20s Sit-up Test (20 s- SU) (Guo et al., 2021).

The participant lay on the yoga mat with his arms crossed in front of his chest. The knee joints were set at 90°  of flexion, with the feet being held on the ground by a researcher. Their elbow should touch the knee joints as the torso flexed, and their scapula should touch the mat as the torso extended. The participants were given verbal encouragement every 5 s. The maximum number that the participants completed in 20 s was recorded.

##### Eight-level abdominal bridge test, 6-level supine bridge test and 5-level side bridge test.

**Figure 1:**
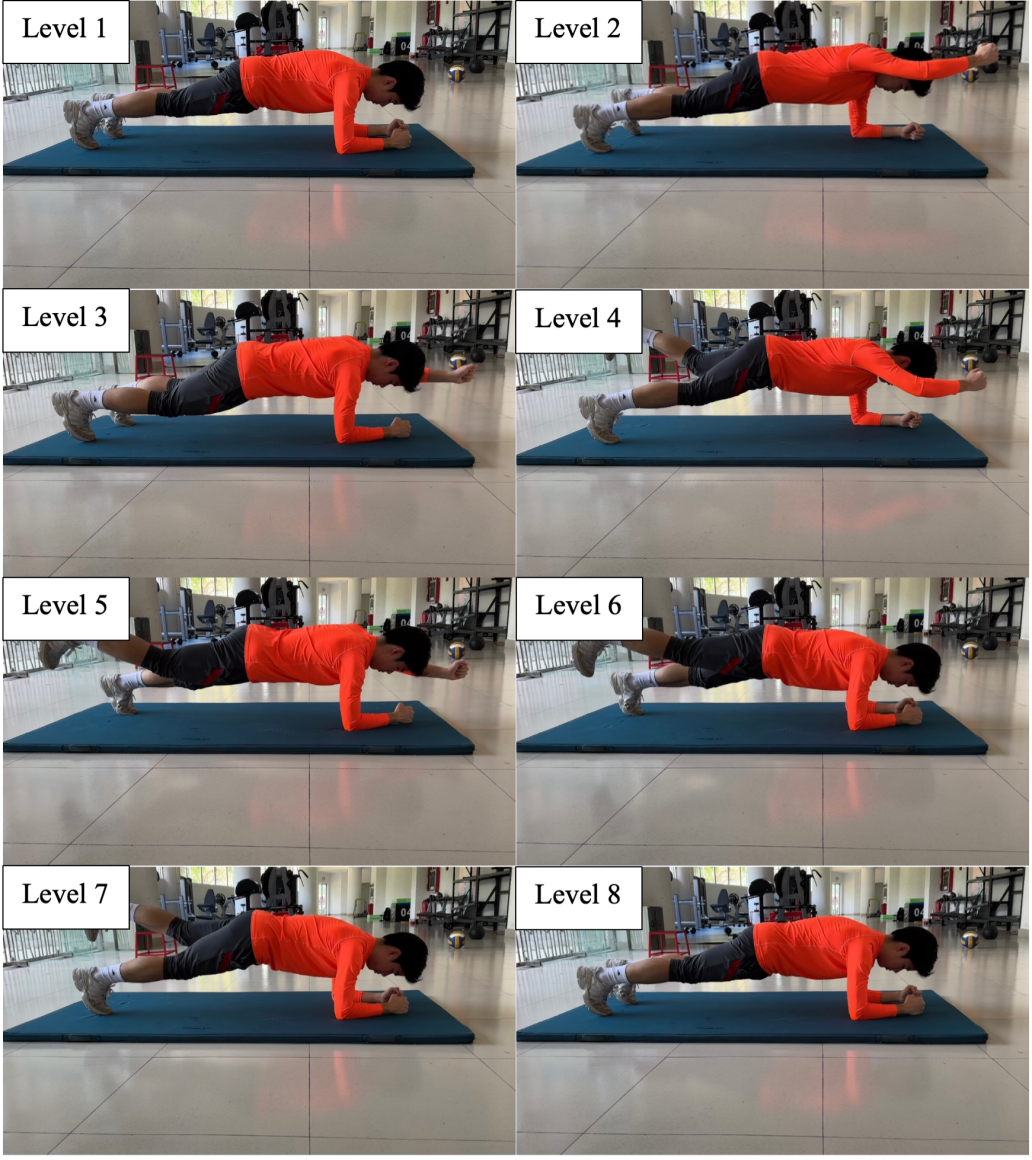
Eight-level abdominal bridge.

Participants were required to complete the 8-level abdominal bridge test, 6-level supine bridge test and 5-level side bridge following the instructions in Table 1, Fig. 1–Fig. 3.

**Table 1:** Eight-level abdominal bridge test, 6-level supine bridge test and 5-level side bridge test (Ting, 2017; Yin, Xiao & Tan, 2016; Shimokochi et al., 2009).

Level	Time	Score	Instructions
8-level abdominal bridge rating			
1	60	1	Participants maintain the plank position with elbows and toes on the ground as the support.
2	15	3	Lift the right arm and extend it until it is parallel to the ground.
3	15	5	Withdraw the right arm and lift the left arm until it is parallel to the ground.
4	15	6	Withdraw the left arm and lift the right leg until it is parallel to the ground.
5	15	10	Lower the right leg and lift the left leg until it is parallel to the ground.
6	15	15	Extend the right arm and hold the left leg straight. Raise the arm higher than the ears.
7	15	25	Lower the right arm and left leg. Raise and straighten the left arm and right leg. The arm must be higher than the ears.
8	30	35	Lower the left arm and right leg and return to the plank position at level 1.
6-level supine bridge rating			
1	30	1	Lie supine on the mat, bring legs together, plantarflex the feet, put the heels on the ground, and clasp the hands in front of the chest.
2	15	3	Lift right leg and keep it parallel to the opposite leg.
3	15	5	Lower the right leg and lift the left leg; keep the left leg parallel to the opposite leg.
4	15	6	Lower the left leg, lift the right leg, and keep it parallel to the opposite leg and swing it outward to the maximum degree.
5	15	10	Lower the right leg, lift the left leg and keep it parallel to the opposite leg, and swing it outward to the maximum degree.
6	30	15	Lower the left leg, return to supine bridge at level 1.
5-level side bridge rating			
1	30	1	Take the elbow and the outside of the foot as support; flex the elbow to 90° degree and place it below the shoulder; put the other hand on the waist; hold up the trunk.
2	15	3	Keep the body straight and lift the non-support leg.
3	15	5	The non-support leg swings forward as much as possible.
4	15	6	The non-support leg swings back as much as possible.
5	15	10	Return to side bridge position at level 1.

**Figure 2:**
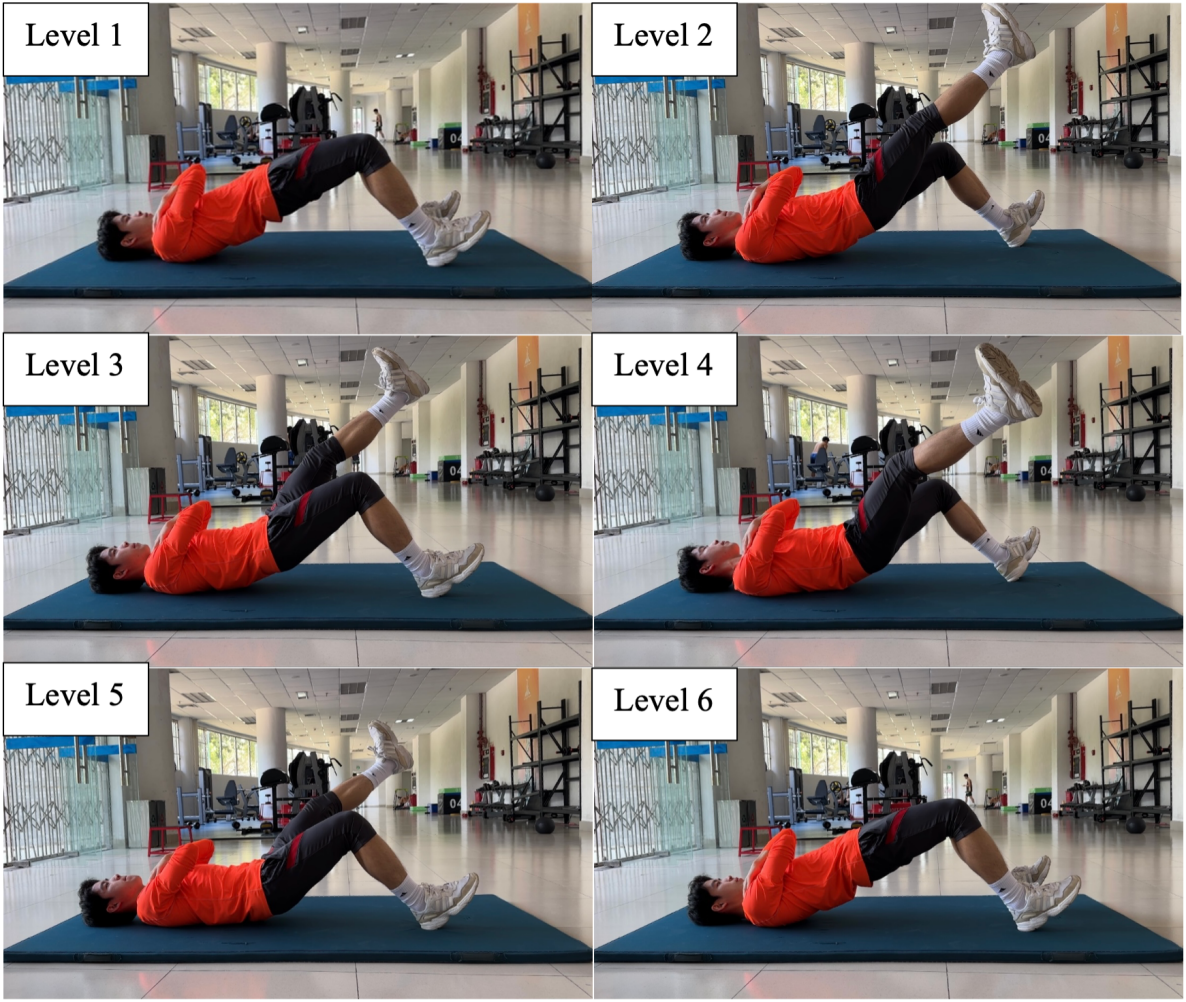
Six-level supine bridge.

**Figure 3:**
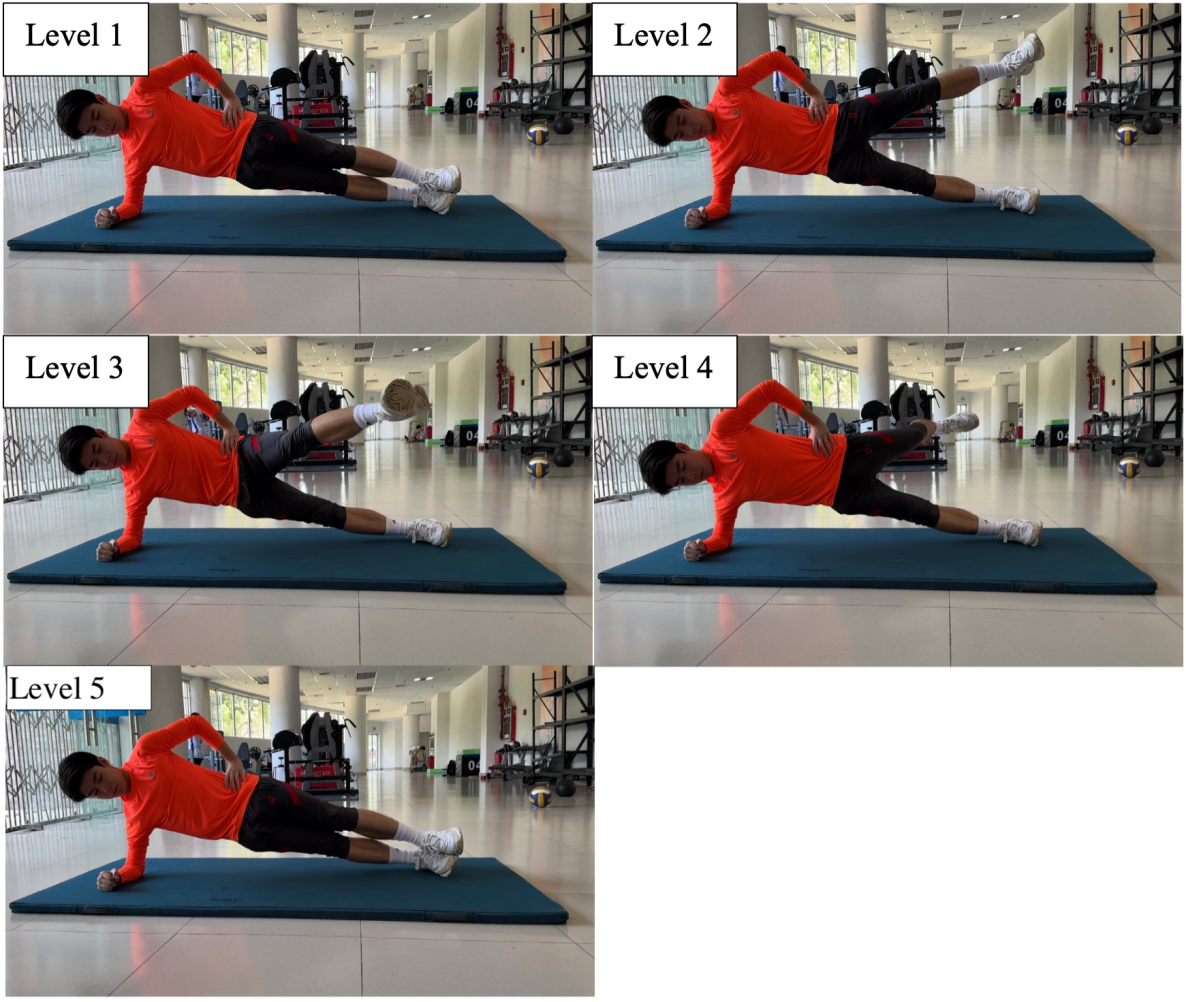
Five-level side bridge.

##### Flexibility test. Trunk ROM test (Guo et al., 2021).

Participants stood barefoot with their feet shoulder-width apart. The distance between their C7 (the 7th cervical vertebra) and S1 (the 1st sacral vertebra) was measured by a tape when the participants were in three postures: for the first posture (P1), the participants maintain a neutral position with arms relaxed; for the second (P2) and third one (P3), they were asked to bend forward and backward as much as possible. The range from P2 to P1 shows torso flexion and from P3 to P1 torso extension.

Hip ROM test (Guo et al., 2021)

Hip flexion was performed from a supine anatomical position, with the knee straight and the hip on the contact surface. Participants lifted their legs voluntarily to the limit. With the greater trochanter of the femur as the joint axis, the hip joint’s ROM was measured by EasyAngle (Meloq AB, Stockholm, Sweden) from the neutral position. Hip extension measurement required participants to lie prone, and the procedure was the same as above. This test was conducted three times, and the results were recorded in degrees. The maximal value was chosen for analysis.

Knee ROM test (Guo et al., 2021)

This test was performed from a prone anatomical position, with knees straight and thighs on the contact surface constantly. Participants bent their knees voluntarily to the limit, and ROM of the knee was measured with the center of the knee joint as the axis by EasyAngle (Meloq AB, Stockholm, Sweden). This test was conducted three times, and the results were recorded in degrees. The maximal value was chosen for analysis.

Ankle ROM test (Guo et al., 2021)

Participants were required to be seated with their knees bent and relaxed, and their feet off the ground without shoes and socks. Participants dorsiflexed and plantarflexed actively to the extreme points. With the lateral malleolus as the joint axis, the researcher measured ROM by EasyAngle (Meloq AB, Stockholm, Sweden). This test was conducted three times, and the results were recorded in degrees. The maximal value was chosen for analysis.

### Skill-related physical fitness test

#### Y-Balance Test

Participants were asked to remove their shoes and socks, and the length of their lower extremities (from the anterior superior iliac spine to the midpoint of the medial malleolus of the ipsilateral foot) was measured and recorded. The participant stood on YBT kit (functionalmovement.com, Danville, VA) with his big toe of dominant foot right behind the red starting line, and hands on his waist. Meanwhile, the participant was required to push the reach indicator as far as possible with the other foot in the front, posteromedial and posterolateral directions, and return to the starting position without losing balance (Zheng et al., 2024). The researcher recorded the maximum distance in the different directions (accurate to 0.5 cm). Each direction had three trials. The average of the three trials in each direction was utilized in the calculation of the results. The Y Balance score = (front + posteromedial + posterolateral)/(3 •lower limb length) •100 (Zheng et al., 2024).

#### Dominant extremity single leg hop distance test (Reid et al., 2007)

Participants hopped and landed on their dominant extremity, and a successful trial was defined as maintaining this landing for at least 2 s. The distance hopped was measured from toe to toe with tape. Three hops were performed, with the longest hop recorded in centimeters for analysis.

### Drop vertical jump test

Participants stood on a 45 cm-high wooden box with their hands on the hips and feet shoulder-width apart. The participant dropped and landed without initial vertical velocity and both feet on the respective force plates. Once landed, he jumped maximally with arms swinging like attempting to rebound and land on the respective force plates again afterward.

Participants were required to wear the same types of shoes to eliminate bias. A Vicon motion capture system (Vicon Metrics Ltd., Oxford, UK) with eight infrared cameras was used to record the motion, with a sampling frequency of 200 Hz. A total of 30 retro-reflective markers, each 14.0 mm in diameter, were attached to bony landmarks according to the Plug-in-Gait model. The makers were placed on all participants by the same researcher, who specialized in biomechanics (McLean et al., 2010). Participants were instructed to stand still on two force plates (AMTI, Watertown, USA) for a static trial to align the joint coordinate system with the laboratory setup before data collection (Leardini et al., 2005). GRF (ground reaction force) was measured by the force plates at a sampling rate of 1,000 Hz, synchronized with the Vicon motion system so that every fifth force data sample matched a single video frame. Static and dynamic calibrations for the force plates and Vicon motion system were conducted as per the manufacturer’s instructions (Guo, Wu & Li, 2020; Guo, Li & Wu, 2018).

The highest trial was selected for further analysis. Data reduction and analysis were performed using Vicon Nexus 2.8.0 software (Vicon Metrics Ltd., Oxford, UK). Both force and kinematic data were filtered using a low-pass, zero-lag Butterworth filter with a cutting-off frequencies of 44 Hz and 14 Hz, respectively (Guo, Wu & Li, 2020). The stance phase of dominant leg was analyzed, defined as the time interval from initial contact with the force plate, when the vertical GRF first exceeded 10 N, to toe-off after the first landing (Bates et al., 2013). Knee moments were reported as external joint moments, calculated using inverse dynamics from the force and kinematic data. The knee angles and angular motions were calculated with 0°  representing the participant’s static standing position.

### Statistical analysis

Statistical analysis was performed with SPSS for Windows (IBM Corp., Armonk, NY, USA). Normal distribution of the data was tested using the Shapiro–Wilk test. Homogeneity of variances was verified by the Levene’s test. The shared variance between the kinetics and kinematics variables of DVJ and physical fitness measurements was estimated using the square of Pearson correlation coefficients (R^2^). Simple linear regression models were first conducted to assess the univariate relationships between each independent variable and the dependent variable. Significant predictors identified in simple linear regression models were then entered simultaneously into a multiple linear regression model to evaluate their collective contribution to the dependent variable. Multicollinearity was assessed using Variance Inflation Factors (VIF), with VIF < 5 indicating acceptable levels. Model fit was evaluated using adjusted R^2^ values. The significance level was set at *p* <  0.05 for all tests. The strength of Pearson correlation coefficients was interpreted as follows: *r* < 0.3 indicated weak, 0.3 ≤*r* < 0.7 moderate, and *r* ≥ 0.7 strong correlations (Portney & Watkins, 2009).

## Results

Table 2 presented the descriptive results of the mean ± standard error (SD) for health-related physical fitness tests, skill-related physical fitness tests and DVJ test respectively.

The univariate linear regression analyses revealed several significant relationships between physical fitness measures and DVJ biomechanics (Table 3). For instance, ankle dorsiflexion ROM was a significant negative predictor of mediolateral ground reaction force (GRFY).

**Table 2:** Descriptive characteristics of the study participants for all physical fitness and DVJ biomechanics variables (*n* = 18).

**Variable**	**Mean ± SD**	**Medin**	**Min**	**Max**
**Health-related physical fitness**				
Core Endurance				
SU (min)	18.6 ± 2.2	18.5	15.0	24.0
8-level abdominal bridge (s)	131.3 ± 38.6	127.5	61.0	180.0
6-level supine bridge (s)	102.4 ± 26.4	120	47.0	120.0
5-level side bridge (s)	55.5 ± 19.4	52.5	33.0	105.0
Flexibility				
Trunk flexion ROM (cm)	13.4 ± 2.2	13.5	9.5	16.9
Trunk extension ROM (cm)	4.9 ± 2.1	4.5	2.0	9.0
Trunk flexion and extension ROM (cm)	18.2 ± 33.3	18.0	13.0	24.4
Hip flexion ROM (°)	80.9 ± 13.4	81.5	55.5	102
Hip extension ROM (°)	24.1 ± 9.4	22.5	13.0	40.0
Knee flexion ROM (°)	129.2 ± 5.7	128.0	119.0	138.0
Ankle dorsiflexion ROM (°)	63.6 ± 8.8	63.0	49.0	82.0
Ankle plantarflexion ROM (°)	142.7 ± 7.4	143.5	129.0	154.0
**Skill-Related Physical Fitness**				
Ya (cm)	66.1 ± 8.6	68.3	48.0	80.0
Ypl (cm)	100.3 ± 6.9	102	87.0	110.5
Ypm (cm)	104.6 ± 4.6	104.3	95.0	114
Y-balance test score	1.02 ± 0.1	1.0	0.9	1.2
DLH (cm)	201.5 ± 18.1	198.8	172.5	233.5
**Drop Vertical Jump Biomechanics**				
Hmax (m)	1.62 ± 0.08	1.6	1.5	1.8
GRFX(N/Kg)	2.94 ± 0.29	2.7	1.5	6.6
GRFY(N/Kg)	2.00 ± 0.39	2.0	1.2	2.7
GRFZ(N/Kg)	16.84 ± 4.77	15.3	10.8	28.1
MhipX (Nm/Kg)	2.35 ± 0.63	2.2	1.3	3.5
MhipY (Nm/Kg)	−1.20 ± 0.48	−1.0	−2.2	−0.6
MhipZ (Nm/Kg)	0.56 ± 0.30	0.5	0.2	1.2
ADhipX (°)	75.6 ± 9.4	78.5	48.3	88.5
ADhipY (°)	6.3 ± 5.3	5.6	0.5	18.4
ADhipZ (°)	26.2 ± 17.1	24.0	2.2	56.1
AhipX (°)	32.3 ± 6. 7	31.0	22.1	44.6
AhipY (°)	4.9 ± 3.7	4.3	0	13.8
AhipZ (°)	9.4 ± 6.3	8.1	0.3	19.2
MkneX (Nm/Kg)	2.3 ± 0.5	2.2	1.5	3.2
ADkneX (°)	103. 1 ± 14.9	104.1	68.3	121.3
AkneX (°)	30.3 ± 10.0	32.6	9.7	43.3
MankX (Nm/Kg)	1.66 ± 0.26	1.6	1.3	2.4
ADankX (°)	34.9 ± 8.7	34.5	21.6	51.7
AankX (°)	13.2 ± 7.6	14.1	0.5	27.5

**Notes.**

SU sit-up ROM range of motion a anterior reach pl posterolateral reach pm posteromedial reach DLH dominant extremity single leg hop distance Hmax maximal center of mass of DVJ GRF ground reactional force X joint flexion and extension angle during first landing of DVJ Y joint adduction and abduction angle during first landing of DVJ Z joint internal and external rotation angle during first landing of DVJ M moment AD angle displacement A angle

**Table 3:** Significant associations from univariate linear regression analyses between physical fitness measures and DVJ biomechanics.

**Independent variable**	**Dependent variable**	**r**	*p*-value	**Regression equation**	**R^2^**
Ankle dorsiflexion ROM	GRFY	−0.48	0.025	GRFY = −0.02141* ankle dorsiflexion ROM+ 3.362	22.9%
5-level side bridge	ADhipY	−0.51	0.014	ADhipY = −0.1387* 5-level side bridge+ 13.96	26.3%
5-level side bridge	MkneX	0.65	0.033	MkneX = 0.015*5-level side bridge	54.7%
8-level abdominal bridge	MkneX	0.59	0.01	MkneX = 0.007*8-level abdominal bridge+ 1.375	35%
Ankle plantarflexion ROM	AkneX	−0.58	0.012	AkneX = −0.7849* ankle plantarflexion ROM+ 142.3	33.6%
Ankle dorsiflexion ROM	ADkneX	−0.64	0.004	ADkneX = −1.087*ankle dorsiflexion ROM+ 172.2	41.2%
Ankle dorsiflexion ROM	MankX	0.59	0.009	MankX = 0.01756* ankle dorsiflexion ROM+ 0.5392	35.3%
Ankle dorsiflexion ROM	ADankX	−0.56	0.015	ADankX = −0.5530* ankle dorsiflexion ROM+ 3.362	31.8%
Trunk flexion ROM	MankX	0.57	0.014	MankX = −0.06609* trunk flexion ROM+ 2.540	32.3%

**Notes.**

ROM range of motion GRF ground reactional force X joint flexion and extension angle during first landing of DVJ Y joint adduction and abduction angle during first landing of DVJ M moment AD angle displacement A angle kne knee joint ank ankle joint

This table summarizes statistically significant correlations (*p* < 0.05). The complete correlation matrix including all correlation coefficients and *p*-values is provided in Tables S1–S4.

In addition to the univariate relationships, multiple linear regression analyses were performed to identify the most parsimonious set of predictors for MkneX and MankX. The model revealed that the 5-level side bridge was a significant predictor while the 8-level abdominal bridge was not retained for MkneX: MkneX = 0.011 × 5-level side bridge score + 1.103 (*p* = 0.004), explaining 45.5% of the variance with no multicollinearity concerns (VIF = 1.296). Another model significantly predicted MankX (*p* = 0.001) and accounted for a great proportion of the variance (*R*^2^ = 61.2%). The regression equation was defined as: MankX = 0.017 × ankle dorsiflexion ROM –0.064 × trunk flexion ROM + 1.426. Both predictors in this model were significant, and variance inflation factors (VIF = 1.001) confirmed the absence of multicollinearity.

## Discussion

This study provides clear evidence regarding the specific research hypotheses that guided our investigation into the relationships between physical fitness and DVJ biomechanics in male collegiate basketball players. There was no significant relationship between skill-related physical fitness tests and DVJ test, however, health-related physical fitness (ankle dorsiflexion ROM, ankle plantarflexion ROM, trunk flexion ROM, 5-level side bridge, 8-level abdominal bridge) related to DVJ biomechanics significantly. Hypothesis 2 was fully supported, with restricted ankle dorsiflexion ROM demonstrating significant correlations with multiple kinetics variables. Hypotheses 1 and 3 received partial support, as core endurance tests showed selective relationships with specific biomechanical parameters, and trunk flexion ROM emerged as a significant predictor of ankle kinetics. Conversely, Hypotheses 4 and 5 were not supported, as the selected skill-related fitness measures showed no significant associations with DVJ biomechanics.

### Relationship between skill-related physical fitness and DVJ

No significant relationship between skill-related physical fitness tests and DVJ test in this study. A study examined the effects of a functional balance training program using unstable surface on lower-limb power in basketball players, as no significant improvements were found in vertical jump performance in either group (Ibrahim et al., 2025). In contrast to plyometric or resistance-based interventions, balance-oriented exercises generally elicit lower activation of high-threshold motor units (Heitkamp et al., 2001; Myer et al., 2006). Although certain studies have reported increases in jump height following instability training among untrained individuals (Behm & Colado, 2012; Boccolini et al., 2013; Sakes et al., 2016), such adaptations may reach a plateau unless supplemented with high-intensity resistance stimuli. Therefore, the relationship between balance and vertical jump performance were not confirmed in this study. As for D-SLHD, it might be incorrect to assume that strength in one direction predicts strength in other directions (Wakai & Linthorne, 2005). Compared to the vertical jump, horizontal jump is a more complex movement and requires the athlete to consider the optimal angle of projection (Meylan et al., 2010). Furthermore, correlation of performance in lower extremity between vertical jump and horizontal jump was lowest, which may be explained by the various demands of the two tests (McGinley et al., 2009; Bertozzi et al., 2024). Therefore, D-SLHD may not be able to predict vertical jump performance.

### Relationship between ankle dorsiflexion ROM and GRFY

Ankle dorsiflexion range of motion (DF ROM) correlated negatively to GRFY. There was a negative relationship between the preservation of balance and stability during straight running and the mediolateral GRF in runners who underwent unilateral transfemoral amputation (Hisano et al., 2021). It was speculated that small mediolateral GRF performed a significant effect of maintaining the balance and stability in DVJ (Gonçalves et al., 2020). Larger DF ROM, with a larger maximum knee angle increased the loading phase duration, which extended the dissipation time of landing forces (Devita & Skelly, 1992), eventually leading to small GRF (Teng, Leong & Kong, 2020) and maintaining the balance and stability during landing (Hisano et al., 2021). In addition, according to the work-energy relationship, downward displacement of the body’s center of mass was increased with greater knee flexion and ankle dorsiflexion, which reduced the force acting on the body (Stefano et al., 2008). Therefore, during the landing of DVJ, with the increased DF ROM, balance and stability improved, and ultimately GRFY decreased. Simultaneously, we observed that the findings from other studies were contrary to ours, which was focused on the preparation of the vertical jump. For example, previous research has indicated a positive correlation between ankle DF ROM and GRFY during the preparation phase of a vertical jump (Koyama, Kato & Yamauchi, 2014). This positive association was primarily explained by the mechanical benefits offered by increased dorsiflexion during the preparatory phase of the vertical jump (Zhang et al., 2024). Enhanced DF ROM allowed for greater forward translation of the tibia, which facilitated deeper knee flexion and greater hip extension (Macrum et al., 2012). Consequently, greater DF ROM was crucial for maximizing the efficiency of force production during vertical jump performance (Fong et al., 2011). Besides, female athletes are particularly susceptible to injuries related to excessive GRF due to differences in neuromuscular control and biomechanical strategies compared to their male counterparts (Myer et al., 2005). Restricted ankle dorsiflexion further exacerbates these risks by limiting the ability to attenuate forces during dynamic movements (Fong et al., 2011). This makes female athletes more prone to injuries, especially during sports involving frequent jumping, cutting, and sudden changes of direction (Myer et al., 2005).

### Relationship between ankle dorsiflexion ROM and ADkneX

Ankle DF ROM correlated negatively to ADkneX. Bell, Padua & Clark (2013) have documented an inverse relationship between ankle DF ROM and ADkneX during dynamic tasks. This relationship is particularly evident in female athletes, who are more prone to movement compensations due to structural and neuromuscular differences compared to their male counterparts (Hewett, Myer & Ford, 2004). For example, Fong et al. (2011) reported that athletes with restricted DF ROM exhibited significantly increased knee flexion angle during landing tasks. The inability of the tibia to advance adequately during dorsiflexion likely forces athletes to compensate for the lack of ankle range of motion by increasing knee flexion angle, resulting in increased angle displacement of knee joint (Macrum et al., 2012). Additionally, Begalle et al. (2015) demonstrated that athletes with greater ankle dorsiflexion exhibited more uniform distribution of ground reaction force, with the pressure primarily concentrated on the forefoot. This redistribution effectively reduced the angle displacement required at the knee joint.

### Relationship between ankle dorsiflexion ROM and ADankX

Ankle DF ROM correlated negatively to ADankX. While direct comparisons for this specific relationship are limited in the literature, its biomechanical plausibility is strongly supported by foundational principles and related findings. The inverse relationship can be explained by the length-tension dynamics of the calf muscles and the Achilles tendon. Begalle et al. (2015) revealed that reduced ankle dorsiflexion was associated with increased knee flexion at initial ground contact during jump landings. Similarly, Ameer & Muaidi (2017) observed that an increase in knee flexion angle during single leg jump landings led to a greater plantarflexion moment. This suggests a negative correlation between ankle dorsiflexion and the displacement of ankle plantarflexion during the landing phase. Furthermore, a key biomechanical principle underlying this negative correlation between dorsiflexion ROM and the displacement of plantarflexion is the length-tension relationship of the calf muscles (gastrocnemius and soleus) and the Achilles tendon (Zellers, Baxter & Silbernagel, 2021). Excessive dorsiflexion can alter the length of these muscles and tendons, reducing their force-generating capacity during the plantarflexion phase (Zellers, Baxter & Silbernagel, 2021). The Achilles tendon, which acts as a spring to store and release elastic energy, also becomes less effective when the ankle joint is held in a dorsiflexed position (Zellers, Baxter & Silbernagel, 2021).

### Relationship between ankle dorsiflexion ROM and MankX

Ankle DF ROM correlated positively to MankX. Increasing DF ROM tends to increase the knee flexion angle (Malloy et al., 2015). Knee flexion angle was proved positively correlated with ankle plantarflexion moment (Ameer & Muaidi, 2017), whose increase could be due to the greater knee angle through the body leaning forward strategy (Macrum et al., 2012). Therefore, it was deduced that ankle DF ROM correlated positively to plantarflexion moment. Moreover, greater dorsiflexion enhances the leverage of the ankle joint, increasing the moment arm and, consequently, the torque generated during plantar flexion (Bell, Padua & Clark, 2013). This increased moment arm facilitates the efficient transmission of force from the lower extremity to the ground, enhancing propulsion (Ameer & Muaidi, 2017). While it has been suggested that this biomechanical advantage is particularly important for injury risk reduction in female athletes (Fong et al., 2011), our results empirically demonstrate that this fundamental relationship between DF ROM and plantarflexion moment is a key feature of dynamic landing in trained male basketball players as well (Fong et al., 2011).

### Relationship between ankle plantarflexion ROM and AkneX

Ankle plantarflexion ROM (PF ROM) correlated negatively to AkneX. A greater capacity for plantarflexion enables a more forefoot-oriented landing posture upon initial contact (Teng, Leong & Kong, 2020). Research showed that foot-landing position significantly affects knee flexion angles at the initial contact during double-leg landing (Zhang, Bates & Dufek, 2000) or single-leg drop landing (Teng, Leong & Kong, 2020). Knee flexion angle at initial contact is smaller at fore foot landings and at natural landings when compared to flat-foot landing (Teng, Leong & Kong, 2020). That is, the restricted plantarflexion is associated with greater knee flexion angles at the initial contact, which is consistent with our result.

### Relationship between trunk flexion ROM and MankX

Trunk flexion ROM correlated negatively with ankle plantarflexion moment during landing. Trunk flexion-extension is an interaction between the lumbar spine and pelvis joints (Blackburn & Padua, 2009). Trunk flexion ROM relied more on pelvic joint instead of lumbar spine (Endo, Miura & Sakamoto, 2020). At the end of trunk flexion, lower pelvic flexibility is associated with greater activation of the hamstrings, which could cause a greater knee flexion angle during functional tasks (Blackburn & Padua, 2009). Meanwhile, the greater knee angle through the body leaning forward strategy increases plantarflexion moment (Blackburn & Padua, 2009), therefore it was deduced that trunk flexibility correlated negatively with plantarflexion moment during landing. The negative relationship can also be explained by the biomechanical role of the trunk in stabilizing and coordinating lower extremity movements. During tasks that involve ankle PF, such as jumping and sprinting, the trunk must maintain a certain degree of flexion to allow the center of mass to move efficiently over the base of support (Willy & Davis, 2011; Kugler & Janshen, 2010). If trunk flexion is restricted, the center of mass shifts posteriorly, leading to an increased demand on the ankle plantar flexors to generate propulsive forces (Willy & Davis, 2011; Davis et al., 2019; Carson, Aslan & Ortega, 2024). This increased demand can result in excessive joint moments at the ankle, which may compromise the efficiency of movement and increase the risk of overuse injuries (Fong et al., 2011). Female athletes are particularly susceptible to these compensatory mechanisms due to their tendency to exhibit reduced neuromuscular control and altered lower extremity kinematics compared to their male counterparts (Myer et al., 2005). The combination of restricted trunk flexion and increased ankle plantar flexion moments can lead to inefficient movement patterns, decreased athletic performance, and an elevated risk of injury, particularly in sports that involve repetitive jumping and landing (Myer et al., 2007). We are not aware of any studies that directly contradict our findings. However, we did identify research demonstrating that, during the vertical jump to takeoff, a proportional relationship between trunk flexion ROM and MankX has been observed. For example, an increased forward trunk lean during the preparatory was phase largely due to the shifting center of mass (Koshino et al., 2024). This forward lean maximized the SSC of the lower limb muscles, including the plantar flexors, leading to enhanced force production during the takeoff phase (Van Hooren & Zolotarjova, 2017; Wilder et al., 2021). Moreover, a study highlighted the importance of trunk mechanics in transferring energy from the upper body to the lower extremities, emphasizing that trunk flexion was key for achieving optimal ankle joint mechanics (Jeong, Choi & Shin, 2021). Therefore, together with ankle dorsiflexion ROM, trunk flexion ROM contributed significantly to MankX.

### Relationship between 5-level side bridge and ADhipY

Five-level side bridge correlated negatively with the ADhipY. Side bridge is an effective core exercise for improving lateral core muscle capacity and thus could reduce the landing error scoring system (LESS) by up to three points (Lin, 2020). LESS was associated with decreased hip adduction angle in the frontal plane during vertical jump (Lin, 2020). Therefore, it was deduced that 5-level side bridge correlated negatively with the hip adduction angle displacement. Furthermore, the negative relationship between 5-level side bridge performance and hip joint adduction can be explained by the biomechanical role of the hip abductors in stabilizing the pelvis and controlling lower extremity alignment during dynamic movements (Hollman et al., 2009). When the hip abductors, particularly the gluteus medius, were weak or fatigued, the pelvis tended to drop on the contralateral side, leading to increased hip adduction on the stance leg (Hollman et al., 2009). This excessive adduction placed additional stress on the knee joint, contributing to valgus collapse and increasing the risk of injury (Hollman et al., 2009).

### Relationship between 5-level side bridge/8-level abdominal bridge and MkneX

Five-level side bridge and 8-level abdominal bridge correlated positively to MkneX. It was reported that both the side bridge and abdominal bridge showed similar muscle activation patterns for the rectus femoris and hamstrings muscles (Abdallah, Mohamed & Hegazy, 2019). The rectus femoris belongs to the quadriceps muscle, which acts as a major knee extensor. The active quadriceps contraction force is a major contributor to the anterior shear force, which is most pronounced at more extended knee joint angles of 0–35 degrees of flexion (Bencke, Aagaard & Zebis, 2018). Whilst hamstring muscle activation at or near full knee extension (0°–30°) produces insufficient posterior force to the leg because of the small angle of inclination of the hamstring tendons (Noyes et al., 2005). The average angle of knee flexion at the initial contact of the ground was 30.28° in this study, at which angle the quadriceps muscle is highly activated and produces maximum anterior shear forces (Noyes et al., 2005). Therefore, it was speculated that these two core endurance tests correlated positively with MkneX. Moreover, the positive relationship between core stability and knee flexion moment can be explained by the biomechanical role of the core muscles in maintaining proper alignment of the pelvis, trunk, and lower limbs (Larwa et al., 2021). When the core is strong and stable, athletes can maintain better control of their trunk and pelvis, reducing excessive forward trunk lean, which has been linked to increased knee flexion and decreased knee valgus (Hewett & Myer, 2005). This control allows for greater knee flexion during landing tasks, which is crucial for dissipating ground reaction forces and preventing injuries (Hewett & Myer, 2005). The lateral core muscles, particularly the gluteus medius, play a key role in stabilizing the pelvis during dynamic movements (Rinaldi et al., 2022). Strong lateral core stability, as developed through exercises like the 5-level side bridge, helps prevent the pelvis from dropping on the contralateral side, which can lead to decreased knee valgus and increased knee flexion (Lin, 2020; Powers, 2010). Similarly, the anterior core muscles activated during the 8-level abdominal bridge contribute to controlling anterior-posterior pelvic tilt, further enhancing knee flexion mechanics. Although the 8-level abdominal bridge was a significant predictor of MkneX in isolation, it did not contribute significantly to the multivariate model when combined with the 5-level side bridge. This suggests that for the practical assessment of MkneX, the 5-level side bridge alone may be a sufficient evaluative tool.

In summary, this study demonstrates that a suite of health-related physical fitness tests, particularly ankle dorsiflexion ROM and core endurance assessments like the 5-level side bridge, are significant correlated to lower extremity biomechanics during the landing phase of a DVJ in male collegiate basketball players. These findings provide empirical support for the practical utility of these field-based measures as accessible proxies for monitoring landing patterns, which may aid in injury risk screening and management. The conclusions, however, must be interpreted within the context of the study’s limitations.

### Practical application

Physical fitness (ankle dorsiflexion ROM, ankle plantarflexion ROM, trunk flexion ROM, 5-level side bridge, 8-level abdominal bridge) related to DVJ biomechanics significantly. Coaches could prioritize these tests to reduce evaluation complexity while retaining biomechanical relevance.

### Limitation

This study focused on male collegiate basketball players. Future work should include female athletes and diverse populations to improve generalizability. Future studies also could incorporate electromyography to explore neuromuscular activation patterns and isokinetic dynamometry to quantify joint-specific strength. Additionally, statistical parametric mapping (SPM) could enhance temporal analysis of kinetic and kinematic waveforms during DVJ. Finally, while some of the identified physical fitness tests cannot elucidate the specific nature of joint involvement, their results are valuable for establishing correlations with jump biomechanics in specific joints.

## Conclusions

This study provides clear evidence regarding the relationships between physical fitness and DVJ biomechanics in male collegiate basketball players, offering specific insights into our research hypotheses. Our findings fully support the crucial role of ankle dorsiflexion ROM (hypotheses 2), which demonstrated comprehensive associations with landing mechanics across multiple kinetic and kinematic variables. The hypotheses concerning core endurance (hypotheses 1) and trunk flexibility (hypotheses 3) received partial support, with specific tests—particularly the 5-level side bridge—showing significant relationships with hip and knee biomechanics. Conversely, our data did not support the predictive value of the skill-related fitness measures (hypotheses 4–5) for landing patterns in this trained population. These results collectively indicate that health-related fitness components, especially ankle dorsiflexion ROM and core endurance, serve as more relevant field-based indicators for monitoring landing biomechanics than the examined skill-related measures. Consequently, coaches and practitioners should prioritize these health-related assessments when developing screening protocols for basketball athletes.

##  Supplemental Information

10.7717/peerj.20613/supp-1 Supplemental Information 1 The raw measurements contain the basic information of each participant and specific data of each test

10.7717/peerj.20613/supp-2 Supplemental Information 2 Supplementary tables Complete correlation between all variables and DVJ biomechanics.

## Additional Information and Declarations

### Data Availability

The following information was supplied regarding data availability: The raw measurements are available in the Supplementary File.
