# Supplementary material for: The relationship between physical fitness and drop vertical jump biomechanics in male college basketball players
Source: PeerJ. 2026 Feb 2;14:e20613. doi: 10.7717/peerj.20613 (PMC12875222; doi:10.7717/peerj.20613)
Supplement: Supplemental Information 2 — Complete correlation between all variables and DVJ biomechanics. [file peerj-14-20613-s002.docx]

Supplementary Table S1. Complete correlation between 20s sit-up test and DVJ biomechanics.

| 20s sit-up test | |
| --- | --- |
| Ground reactional force (r, p) | |
| GRFX | 0.13, 0.604 |
| GRFY | -0.27, 0.282 |
| GRFZ | 0.30, 0.222 |
| Hip joint (r, p) | |
| MhipX | -0.01, 0.980 |
| MhipY | 0.09, 0.716 |
| MhipZ | 0.30, 0.227 |
| ADhipX | -0.24, 0.337 |
| ADhipY | 0.15, 0.550 |
| ADhipZ | 0.25, 0.310 |
| AhipX | 0.002, 0.994 |
| AhipY | -0.02, 0.938 |
| AhipZ | 0.19, 0.444 |
| Knee joint (r, p) | |
| MkneX | 0.48, 0.440 |
| MkneY | 0.38, 0.121 |
| MkneZ | -0.34, 0.168 |
| ADkneX | 0.03, 0.922 |
| ADkneY | -0.17, 0.503 |
| ADkneZ | 0.12, 0.637 |
| AkneX | 0.13, 0.620 |
| AkneY | -0.17, 0.502 |
| AkneZ | -0.04, 0.877 |
| Ankle joint (r, p) | |
| MankX | 0.21, 0.410 |
| MankY | -0.04, 0.878 |
| MankZ | -0.12, 0.635 |
| ADankX | 0.27, 0.276 |
| ADankY | 0.28, 0.266 |
| ADankZ | -0.22, 0.386 |
| AankX | -0.46, 0.053 |
| AankY | -0.34, 0.167 |
| AankZ | 0.20, 0.438 |

Notes: GRF: ground reactional force; X: joint flexion and extension angle during first landing of DVJ; Y: joint adduction and abduction angle during first landing of DVJ; Z: joint internal and external rotation angle during first landing of DVJ; M: moment; AD: angle displacement; A: angle; ap < 0.05; bp < 0.01.

Supplementary Table S2. Complete correlation between bridge tests and DVJ biomechanics.

|  | 8-level abdominal bridg(s) | 6-level supine bridge(s) | 5-level side bridge(s) |
| --- | --- | --- | --- |
| Ground reactional force (r, p) | | | |
| GRFX | 0.02, 0.951 | -0.39, 0.111 | -0.2, 0.439 |
| GRFY | -0.08, 0.766 | -0.22, 0.371 | -0.35, 0.156 |
| GRFZ | 0.49, 0.370 | -0.13, 0.609 | 0.11, 0.674 |
| Hip joint (r, p) | | | |
| MhipX | 0.17, 0.514 | -0.15, 0.545 | -0.11, 0.670 |
| MhipY | 0.01, 0.971 | 0.46, 0.057 | 0.24, 0.337 |
| MhipZ | 0.23, 0.357 | -0.4, 0.101 | 0.22, 0.389 |
| ADhipX | 0.09, 0.719 | 0.21, 0.396 | 0.03, 0.901 |
| ADhipY | -0.34, 0.163 | -0.36, 0.149 | -0.51a, 0.030 |
| ADhipZ | 0.07, 0.769 | 0.01, 0.985 | 0.02, 0.935 |
| AhipX | 0.17, 0.511 | 0.16, 0.537 | 0.06, 0.815 |
| AhipY | -0.01, 0.969 | -0.1, 0.689 | -0.13, 0.615 |
| AhipZ | 0.22, 0.374 | -0.16, 0.536 | 0.14, 0.583 |
| Knee joint (r, p) | | | |
| MkneX | 0.59b, 0.01 | 0.21, 0.398 | 0.65a, 0.004 |
| MkneY | 0.27, 0.279 | 0.03, 0.905 | 0.19, 0.461 |
| MkneZ | -0.16, 0.535 | -0.12, 0.648 | -0.22, 0.388 |
| ADkneX | -0.03, 0.908 | 0.22, 0.374 | -0.15, 0.548 |
| ADkneY | -0.47, 0.470 | -0.06, 0.824 | -0.37, 0.131 |
| ADkneZ | 0.11, 0.659 | -0.06, 0.827 | -0.21, 0.411 |
| AkneX | 0.11, 0.671 | -0.06, 0.822 | 0.09, 0.715 |
| AkneY | 0.13, 0.602 | 0.31, 0.215 | 0.03, 0.897 |
| AkneZ | -0.17, 0.495 | -0.43, 0.074 | -0.35, 0.156 |
| Ankle joint (r, p) | | | |
| MankX | 0.21, 0.399 | -0.26, 0.308 | 0.21, 0.408 |
| MankY | 0.12, 0.624 | -0.03, 0.919 | -0.12, 0.630 |
| MankZ | 0.11, 0.672 | -0.07, 0.771 | -0.08, 0.740 |
| ADankX | -0.12, 0.632 | 0.14, 0.593 | -0.22, 0.390 |
| ADankY | -0.1, 0.707 | -0.04, 0.885 | -0.05, 0.836 |
| ADankZ | -0.10, 0.986 | -0.09, 0.713 | -0.25, 0.320 |
| AankX | -0.45, 0.060 | -0.1, 0.681 | -0.54, 0.020 |
| AankY | -0.29, 0.247 | -0.13, 0.597 | -0.25, 0.317 |
| AankZ | 0.21, 0.395 | 0.02, 0.938 | -0.02, 0.927 |

Notes: GRF: ground reactional force; X: joint flexion and extension angle during first landing of DVJ; Y: joint adduction and abduction angle during first landing of DVJ; Z: joint internal and external rotation angle during first landing of DVJ; M: moment; AD: angle displacement; A: angle; ap < 0.05; bp < 0.01.

Supplementary Table S3. Complete correlation between flexibility tests and DVJ biomechanics.

|  | Trunk flexion ROM | Trunk extension ROM | Trunk flexion and extension ROM | Hip flexion ROM | Hip extension ROM | Knee flexion ROM | Ankle dorsiflexion ROM | Ankle plantarflexion ROM |
| --- | --- | --- | --- | --- | --- | --- | --- | --- |
| Ground reactional force (r, p) | | | | | | | | |
| GRFX | -0.18, 0.485 | 0.32, 0.201 | 0.08, 0.761 | 0.11, 0.660 | -0.21, 0.408 | 0.15, 0.566 | 0.35,  0.155 | 0.01,  0.969 |
| GRFY | -0.1, 0.703 | -0.2, 0.427 | -0.19, 0.456 | -0.20, 0.417 | -0.06, 0.802 | -0.12, 0.633 | -0.48^a^, 0.045 | 0.23,  0.355 |
| GRFZ | -0.18, 0.468 | 0.19, 0.461 | -0.01, 0.974 | 0.26, 0.308 | 0.04, 0.888 | -0.02, 0.931 | 0.35,  0.161 | -0.06,  0.826 |
| Hip joint (r, p) | | | | | | | | |
| MhipX | -0.28, 0.258 | 0.06, 0.825 | -0.15, 0.542 | -0.32, 0.195 | -0.49, 0.410 | -0.04, 0.873 | 0.51,  0.290 | 0.03,  0.893 |
| MhipY | 0.23, 0.353 | -0.2, 0.430 | 0.03, 0.894 | 0.30, 0.225 | 0.12, 0.625 | -0.28, 0.253 | -0.07,  0.795 | -0.46,  0.056 |
| MhipZ | -0.23, 0.363 | -0.09, 0.725 | -0.21, 0.408 | -0.35, 0.156 | -0.11, 0.673 | -0.17, 0.501 | 0.33,  0.176 | 0.18,  0.472 |
| ADhipX | 0.37, 0.127 | 0.09, 0.732 | 0.30, 0.220 | -0.11, 0.677 | 0.35, 0.153 | 0.22, 0.375 | -0.25,  0.309 | -0.11,  0.655 |
| ADhipY | 0.09, 0.712 | -0.31, 0.211 | -0.13, 0.612 | -0.21, 0.40 | -0.26, 0.293 | -0.09, 0.711 | 0.01,  0.968 | 0.12,  0.632 |
| ADhipZ | -0.2, 0.438 | -0.21, 0.412 | -0.26, 0.302 | -0.12, 0.633 | -0.26, 0.301 | 0.32, 0.202 | -0.36,  0.147 | 0.29,  0.238 |
| AhipX | -0.19, 0.458 | 0.25, 0.319 | 0.03, 0.912 | 0.12, 0.650 | 0.14, 0.570 | 0.11, 0.656 | 0.02,  0.953 | -0.48,  0.440 |
| AhipY | -0.02, 0.936 | -0.1, 0.686 | -0.08, 0.767 | 0.12, 0.627 | 0.32, 0.193 | 0.14, 0.590 | -0.32,  0.202 | -0.35,  0.159 |
| AhipZ | -0.48 0.430 | -0.47, 0.051 | -0.61, 0.077 | -0.27, 0.270 | -0.39, 0.113 | -0.03, 0.895 | 0.33, 0.179 | 0.1,  0.706 |
| Knee joint (r, p) | | | | | | | | |
| MkneX | -0.36, 0.142 | 0.01, 0.979 | -0.24, 0.342 | -0.02, 0.945 | -0.05, 0.843 | 0.24, 0.347 | 0.42,  0.082 | -0.16,  0.521 |
| MkneY | -0.29, 0.240 | -0.07, 0.777 | -0.24, 0.338 | 0.10, 0.708 | -0.08, 0.768 | -0.08, 0.740 | 0.5,  0.330 | 0.25,  0.320 |
| MkneZ | 0.13, 0.609 | 0.19, 0.463 | 0.20, 0.425 | 0.04, 0.861 | -0.19, 0.456 | 0.41, 0.088 | -0.06,  0.827 | -0.09,  0.731 |
| ADkneX | 0.35, 0.150 | 0.16, 0.516 | 0.34, 0.170 | 0.08, 0.762 | 0.21, 0.406 | 0.26, 0.301 | -0.64^b^, 0.004 | 0.07,  0.772 |
| ADkneY | 0.31, 0.212 | 0.35, 0.158 | 0.42, 0.082 | 0.33, 0.176 | 0.19, 0.443 | 0.24, 0.331 | -0.42,  0.082 | 0.04,  0.863 |
| ADkneZ | 0.31, 0.992 | 0.13, 0.608 | 0.08, 0.758 | 0.14, 0.594 | 0.29, 0.243 | -0.21, 0.411 | -0.19,  0.450 | 0.09,  0.732 |
| AkneX | -0.16, 0.536 | 0.06, 0.804 | -0.07, 0.795 | -0.16, 0.536 | -0.08, 0.761 | 0.37, 0.126 | 0.05,  0.844 | -0.58^a^,  0.012 |
| AkneY | 0.17, 0.509 | 0.34, 0.166 | 0.32, 0.193 | 0.62, 0.060 | 0.53, 0.250 | -0.02, 0.944 | -0.41,  0.092 | -0.12,  0.645 |
| AkneZ | -0.08, 0.754 | 0.13, 0.599 | 0.03, 0.911 | -0.33, 0.179 | 0.02, 0.954 | 0.13, 0.615 | 0.14,  0.584 | -0.08,  0.745 |
| Ankle joint (r, p) | | | | | | | | |
| MankX | -0.57^a^, 0.014 | -0.14, 0.569 | -0.47, 0.490 | -0.2, 0.417 | -0.24, 0.341 | 0.01, 0.982 | 0.59^a^,  0.009 | 0.06,  0.812 |
| MankY | -0.16, 0.526 | -0.05, 0.857 | -0.14, 0.591 | 0.23, 0.366 | -0.32, 0.198 | -0.22, 0.393 | 0.26,  0.290 | -0.07,  0.787 |
| MankZ | 0.3, 0.227 | 0.18, 0.479 | 0.31, 0.209 | 0.1, 0.682 | -0.09, 0.726 | 0.24, 0.340 | -0.01,  0.969 | -0.13,  0.604 |
| ADankX | 0.07, 0.782 | 0.16, 0.525 | 0.15, 0.563 | 0.1, 0.697 | -0.13, 0.608 | 0.27, 0.276 | -0.56^b^, 0.015 | 0.31,  0.204 |
| ADankY | -0.15, 0.541 | -0.2, 0.425 | -0.23, 0.365 | 0.31, 0.212 | -0.06, 0.816 | -0.08, 0.746 | -0.08,  0.759 | -0.01,  0.960 |
| ADankZ | 0.15, 0.564 | -0.21, 0.412 | -0.03, 0.908 | -0.28, 0.269 | -0.26, 0.294 | -0.53, 0.250 | 0.22,  0.375 | -0.13,  0.595 |
| AankX | 0.26, 0.294 | 0.07, 0.780 | 0.22, 0.382 | -0.39, 0.114 | -0.48, 0.450 | 0.11, 0.655 | -0.15,  0.554 | 0.35,  0.150 |
| AankY | 0.12, 0.625 | 0.08, 0.746 | 0.13, 0.597 | -0.21, 0.396 | -0.18, 0.466 | 0.34, 0.170 | -0.31,  0.208 | -0.3,  0.234 |
| AankZ | 0.11, 0.679 | -0.24, 0.341 | -0.08, 0.763 | -0.34, 0.171 | -0.42, 0.087 | -0.52, 0.270 | 0.34,  0.162 | 0.09,  0.735 |

Notes: GRF: ground reactional force; X: joint flexion and extension angle during first landing of DVJ; Y: joint adduction and abduction angle during first landing of DVJ; Z: joint internal and external rotation angle during first landing of DVJ; M: moment; AD: angle displacement; A: angle; a^p^ < 0.05; b^p^ < 0.01.

Supplementary Table S4. Complete correlation between Y-balance test and DVJ biomechanics.

| Y-balance test | | | | | DLH |
| --- | --- | --- | --- | --- | --- |
|  | Ya | Ypm | Ypl | Y-score |  |
| Ground reactional force (r, p) | | | | | |
| GRFX | -0.06, 0.810 | -0.29, 0.252 | -0.37, 0.136 | -0.20, 0.422 | -0.41, 0.561 |
| GRFY | -0.18, 0.488 | 0.02, 0.943 | 0.12, 0.647 | -0.06, 0.822 | 0.29, 0.824 |
| GRFZ | -0.18, 0.471 | -0.39, 0.109 | -0.27, 0.274 | 0.00, 0.997 | -0.20, 0.114 |
| Hip joint (r, p) | | | | | |
| MhipX | -0.08, 0.756 | -0.03, 0.907 | -0.41, 0.091 | -0.47, 0.052 | -0.09, 0.068 |
| MhipY | -0.17, 0.512 | -0.16, 0.519 | -0.10, 0.689 | 0.23, 0.360 | -0.12, 0.298 |
| MhipZ | 0.13, 0.619 | 0.14, 0.569 | -0.06, 0.824 | -0.32, 0.191 | 0.28, 0.392 |
| ADhipX | 0.27, 0.271 | 0.47, 0.050 | 0.39, 0.112 | 0.18, 0.472 | 0.29, 0.638 |
| ADhipY | -0.06, 0.819 | -0.17, 0.502 | 0.00, 0.991 | 0.10, 0.697 | -0.27, 0.356 |
| ADhipZ | 0.08, 0.745 | 0.15, 0.556 | 0.17, 0.495 | 0.00, 0.995 | 0.39, 0.490 |
| AhipX | 0.07, 0.785 | 0.38, 0.125 | 0.13, 0.610 | 0.18, 0.481 | 0.00, 0.472 |
| AhipY | -0.07, 0.797 | 0.18, 0.478 | 0.41, 0.092 | 0.39, 0.114 | -0.04, 0.799 |
| AhipZ | -0.08, 0.758 | -0.04, 0.870 | -0.06, 0.805 | -0.24, 0.330 | 0.31, 0.190 |
| Knee joint (r, p) | | | | | |
| MkneX | 0.16, 0.539 | -0.02, 0.936 | -0.16, 0.515 | -0.10, 0.681 | 0.36, 0.230 |
| MkneY | -0.09, 0.714 | -0.43, 0.076 | -0.18, 0.471 | -0.06, 0.824 | -0.21, 0.059 |
| MkneZ | 0.39, 0.106 | 0.58, 0.110 | 0.44, 0.065 | 0.07, 0.795 | 0.24, 0.605 |
| ADkneX | 0.16, 0.533 | 0.28, 0.258 | 0.31, 0.210 | 0.35, 0.152 | 0.10, 0.322 |
| ADkneY | 0.11, 0.659 | 0.15, 0.549 | -0.04, 0.878 | 0.11, 0.654 | -0.27, 0.310 |
| ADkneZ | -0.04, 0.864 | -0.13, 0.606 | 0.06, 0.818 | 0.24, 0.332 | -0.11, 0.925 |
| AkneX | 0.11, 0.679 | 0.37, 0.129 | -0.13, 0.616 | -0.14, 0.588 | 0.17, 0.252 |
| AkneY | -0.18, 0.483 | 0.08, 0.751 | 0.20, 0.417 | 0.34, 0.167 | -0.20, 0.152 |
| AkneZ | -0.21, 0.412 | -0.20, 0.417 | -0.30, 0.222 | -0.32, 0.192 | -0.33, 0.328 |
| Ankle joint (r, p) | | | | | |
| MankX | 0.10, 0.691 | 0.03, 0.918 | -0.03, 0.893 | -0.18, 0.474 | 0.07, 0.210 |
| MankY | -0.04, 0.887 | -0.01, 0.980 | 0.01, 0.981 | 0.08, 0.748 | -0.04, 0.565 |
| MankZ | 0.25, 0.323 | 0.20, 0.416 | 0.00, 0.986 | 0.06, 0.804 | 0.19, 0.903 |
| ADankX | 0.12, 0.647 | 0.13, 0.617 | 0.00, 0.991 | 0.11, 0.652 | 0.09, 0.601 |
| ADankY | -0.01, 0.956 | -0.10, 0.701 | 0.20, 0.435 | 0.25, 0.324 | -0.04, 0.880 |
| ADankZ | -0.21, 0.410 | -0.09, 0.735 | 0.11, 0.665 | -0.14, 0.581 | -0.08, 0.714 |
| AankX | 0.01, 0.982 | -0.10, 0.706 | -0.09, 0.734 | -0.26, 0.296 | -0.15, 0.312 |
| AankY | -0.02, 0.947 | 0.11, 0.653 | -0.16, 0.517 | -0.20, 0.428 | 0.04, 0.295 |
| AankZ | -0.13, 0.596 | -0.15, 0.561 | -0.19, 0.456 | -0.26, 0.294 | 0.01, 0.631 |

Notes: GRF: ground reactional force; X: joint flexion and extension angle during first landing of DVJ; Y: joint adduction and abduction angle during first landing of DVJ; Z: joint internal and external rotation angle during first landing of DVJ; M: moment; AD: angle displacement; A: angle; a: anterior reach; pl: posterolateral reach; pm: posteromedial reach; DLH: dominant extremity single leg hop distance; ap < 0.05; bp < 0.01.
